# A Treatise on General Pathology

**Published:** 1854-03

**Authors:** 


### EDITORIAL.

*A Treatise on General Pathology*; by Dr. J. Henle, Professor of Anatomy and Physiology in Heidelberg. Translated from the German by Henry C. Preston, A. M., M. D. Philadelphia : Lindsay and Blackiston, 1853.

*Principles of Medicine:* comprising General Pathology and Therapeutics, and a brief general view of Etiology, Nosology, Semeiolagy, Diagnosis, Prognosis and Hygienics ; by Charles J. B. Williams, M. D., F. R. S., &c., &c. Edited, with additions, by Meredith Clymer, M. D., &c., &c. Fourth American edition. Philadelphia : Lee and Blanchard, 1853.

THE two works, the titles of which are given above, may be taken as able and extremely valuable exponents of two essentially different classes of investigators.

The first is the production of an eminent German scholar, and is an interesting representation of the *theoretical* or transcendental tendencies of medical philosophy.

The latter is the production of an equally accomplished English scholar and physician, and represents, with equal truth, the "matter of fact," empyrical and inductive tendencies of modern pathology. The first, in his introduction, says : "Two systems contend for the superiority, which we must compare more carefully than has ever been done, and which we must separate more rigidly than has ever been possible in practice, in order to know the value of each, and to establish peace between the champions of both sides." These two systems he describes as follows, viz. : "One system, by consciously abandoning all investigation into the *first cause* and the internal connection of symptoms, describes the form of disease according to the external phenomena : its descriptions furnish only substitutes for sensible impressions: its names are not definitions, but only *nomina propria*, and are so much the more welcome the less a definite idea attaches to them : and should it need a word, for example, inflammation, it designates thereby nothing but the association of "redness, heat, tumefaction and pain."

"The *second* system, which has been called the Theoretical, Physiological, or Rational, endeavors to take up the symptoms in their dependence on each other, and in their connexion with internal changes, and considers these changes as the consequences of external influences upon the organic matter which is endowed with its own inherent vital powers. The causes which it thinks it has discovered as the occasion of the symptoms, this system substitutes for the symptoms where it describes diseases; it loves to note the exact direction which the operation of morbid changes takes, and exchanges, for instance, the name, 'inflammation,' for such names as 'increased plasticity,' 'hyperæmia,' 'stasis' and others. Again, theoretical medicine searches after the absolute nature and power of the remedy, and after the so-called physiological effect, that is, the mode and manner by which the substance and vital power of the organism is altered." After comparing these two systems, which he styles Empyrical and Rational, at considerable length, he concludes with the following just remarks:

"We conclude—that as pure empiricism is not sufficient for all cases, and only in a very few can be our sole therapeutic guide: as we cannot help proceeding, in particular cases, according to particular reflections, which can only arise from our views of the importance of the symptoms and the nature of the remedy: so it is our duty to search as deeply as possible into the nature and internal connection of the phenomena in which we shall interfere.

I think I have demonstrated in the foregoing why there is a rigid separation of the empirical and the rational systems, which, as I remarked in the beginning, cannot be carried out in reality. The rude characteristics of both which we have sketched, suffices to show in how intimate and indissoluble a connection they stand, and we demand only what both parties tacitly, and even contrary to their expressed assurance, have already accomplished, when we expect every one to be at the same time empiric and theorist. Therefore, should not only the mutual deficiencies of empirical and theoretical medicine be supplied, but both should be reciprocally promoted where they can be simultaneously applied."

After thus defining the empirical and rational systems and their relations to each other, Dr. Henle proceeds in his introduction to give a brief and interesting, though very imperfect, history of medical systems from the earliest ages to the present time. The body of the work, which is intended to be a complete treatise on *General Pathology*, is embraced in four chapters, with the following titles, viz:

"1st. The Inquiry into the Idea and Nature of Disease.

"2nd. The Doctrine of the Causes of Disease in General, or General Etiology.

"3d. The Local Relations of Disease; the Conditions of its Propagation in the Organism; the Manner of its Transition from one Organ to another.

"4th. The Relations of Disease in regard to Time, or the General History of Disease, its course, Duration and Termination."

The third chapter occupies a large part of the book, and chiefly relates to the local relations of disease and the mode of its propagation from one organ or tissue to another, until it involves, more or less, the whole system. Dr. Henle evidently regards the nervous system as the principal medium through which the organs are placed in relation with each other, both in health and disease. Hence, a discussion of the healthy and morbid, or "normal and abnormal," *sympathies*, through the various divisions of the nervous system, constitutes by far the most prominent part of the work. Only three or four pages are devoted to what the author styles "normal or healthy sympathies through the medium of the blood." And less than half of that space to the "abnormal sympathies" through the same medium. We have looked through this work in vain to find a clear analysis of those complex morbid phenomena that constitute the great leading classes of disease, or that concise statement of facts in relation to the changes which take place in the solids and fluids of the system, that the reputation of the author had led us to expect. On the contrary, much of the book is written in vague and indefinite language, requiring repeated reading to get fully the meaning of the author. This may be, in part, the fault of the translator. Still, the work of Dr. Henle may be read with interest and profit, both by the student and the parctitioner.

The "Principles of Medicine," by Dr. Williams, is too well known o require any extended analysis at this time. In its general avoidance of mere speculation, its careful collection of facts, and its detailed account of the changes in the composition and movements of the blood, it contrasts strongly with the work of Dr. Henle. The work of Dr. Williams embraces a pretty full consideration of Etiology, Pathology Proper, Semeiology, Diagnosis, Prognosis and a brief view of Hygienics. Under the second head, the author enters upon the inquiry into the nature of diseases, by first considering the elements that enter into their composition; thereby adopting the only philosophical mode of investigating complex morbid phenomena. And we have no hesitation in recommending the "Principles of Medicine" as the best text-book on General Pathology that we possess. Still it is defective in several important particulars. For instance, in speaking of the primary elements of disease, the author makes no distinction between those *elementary properties* which are common to all living matter, and those primary *functions* which belong to particular elementary structures. Indeed, he seems not to have recognized the existence of the former in any way as properties distinct from the primary functions. This omission almost necessarily led to one or two others of still greater importance. Hence, in considering the *elements* of that complex morbid process which we call inflammation, he enumerates only the changes in the movements and composition of the blood, without any reference to the coincident changes in the properties of the solids. His pathology of inflammation is consequently too exclusively humoral; not because he has invested the changes in the movements and composition of the blood with undue importance, but from the omission to place along with them the equally essential and primary changes in the properties of the tissue which constitutes the seat of disease. The same failure to recognize and clearly define the elementary properties of living matter, led to the entire omission of any attempt to analyze the phenomena of idiopathic fevers, or to explain their pathology in any manner. This constitutes the *great* defect of the work ; and one that will be felt and regretted by every careful reader. The analytical method adopted by Dr. Williams is the only successful method of cultivating pathology, whether general or special; and notwithstanding the defects which we have pointed out, we again commend his work to the patronage of the profession. Tne additions made by the editor, Dr. Clymer, are judicious and valuable.

